# A systematic comparison of pharmacogene star allele calling bioinformatics algorithms: a focus on *CYP2D6* genotyping

**DOI:** 10.1038/s41525-020-0135-2

**Published:** 2020-08-03

**Authors:** David Twesigomwe, Galen E. B. Wright, Britt I. Drögemöller, Jorge da Rocha, Zané Lombard, Scott Hazelhurst

**Affiliations:** 1 Sydney Brenner Institute for Molecular Bioscience, University of the Witwatersrand, Johannesburg, South Africa; 2 Division of Human Genetics, National Health Laboratory Service, and School of Pathology, Faculty of Health Sciences, University of the Witwatersrand, Johannesburg, South Africa; 3 Neuroscience Research Program, Kleysen Institute for Advanced Medicine, Winnipeg Health Sciences Centre and Max Rady College of Medicine, University of Manitoba, Winnipeg, MB Canada; 4 Department of Pharmacology and Therapeutics, Rady Faculty of Health Sciences, University of Manitoba, Winnipeg, MB Canada; 5 Department of Biochemistry and Medical Genetics, Rady Faculty of Health Sciences, University of Manitoba, Winnipeg, MB Canada; 6 School of Electrical and Information Engineering, University of the Witwatersrand, Johannesburg, South Africa

**Keywords:** Genome informatics, Pharmacogenomics, Haplotypes

## Abstract

Genetic variation in genes encoding cytochrome *P450* enzymes has important clinical implications for drug metabolism. Bioinformatics algorithms for genotyping these highly polymorphic genes using high-throughput sequence data and automating phenotype prediction have recently been developed. The *CYP2D6* gene is often used as a model during the validation of these algorithms due to its clinical importance, high polymorphism, and structural variations. However, the validation process is often limited to common star alleles due to scarcity of reference datasets. In addition, there has been no comprehensive benchmark of these algorithms to date. We performed a systematic comparison of three star allele calling algorithms using 4618 simulations as well as 75 whole-genome sequence samples from the GeT-RM project. Overall, we found that Aldy and Astrolabe are better suited to call both common and rare diplotypes compared to Stargazer, which is affected by population structure. Aldy was the best performing algorithm in calling *CYP2D6* structural variants followed by Stargazer, whereas Astrolabe had limitations especially in calling hybrid rearrangements. We found that ensemble genotyping, characterised by taking a consensus of genotypes called by all three algorithms, has higher haplotype concordance but it is prone to ambiguities whenever complete discrepancies between the tools arise. Further, we evaluated the effects of sequencing coverage and indel misalignment on genotyping accuracy. Our account of the strengths and limitations of these algorithms is extremely important to clinicians and researchers in the pharmacogenomics and precision medicine communities looking to haplotype *CYP2D6* and other pharmacogenes using high-throughput sequencing data.

## Introduction

Genetic variation is known to influence the way in which individuals respond to therapeutics. Groups of variants that are inherited together, known as haplotypes, provide a basis for phenotype prediction and treatment decisions during pharmacogenomic testing. Accurately detecting functional haplotypes, popularly known as star (*) alleles, in clinically actionable pharmacogenes (e.g. cytochrome *P450* genes) is therefore crucial to the implementation of personalised medicine. In addition, determining pharmacogene star allele frequencies and consequently predicting the phenotypic landscape is a crucial part of large-scale population genetic studies, which can help inform drug policy^1^.

Of the clinically relevant pharmacogenes, *CYP2D6* is one of the most widely studied owing to its contribution to the Phase 1 metabolism of ~25% of clinically prescribed drugs, and high genetic variability within and between populations^2^. Drugs metabolised by *CYP2D6* include various antidepressants, antipsychotics, anticancer agents (e.g. tamoxifen), and opioids (https://drug-interactions.medicine.iu.edu/MainTable.aspx, last accessed: 19-02-2020).

**Fig. 1:**
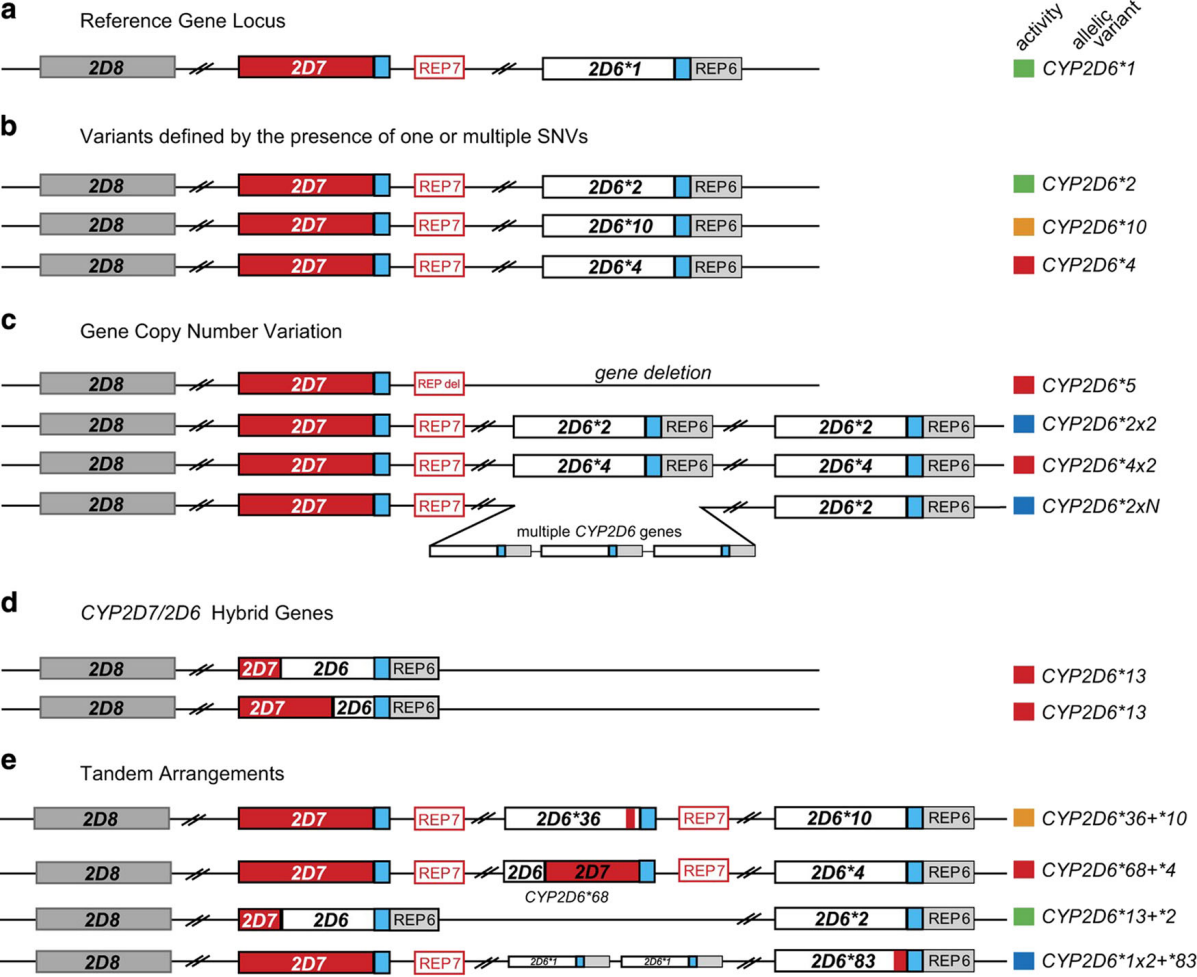
Graphical overview of the highly polymorphic *CYP2D6/2D7/2D8* locus, by Twist et al.^15^, licensed under Creative Commons CC BY 4.0 (https://creativecommons.org/licenses/by/4.0/). No changes have been made to the figure content. **a** The relative position of the reference CYP2D6*1 haplotype (white) to two non-functional paralogs, *CYP2D7* (red) and *CYP2D8* (grey) on the minus strand of Chromosome 22. REP6 and REP7 are paralogous, Alu-containing, 600-bp repetitive sequences found downstream of *CYP2D6* and *CYP2D7*, respectively. The blue boxes indicate identical unique sequences downstream of *CYP2D6* and *CYP2D7*. Notice the “spacer” (1.6-kb) separating REP7 from *CYP2D7* but none between *CYP2D6* and REP6. **b** Common *CYP2D6* star alleles defined by core single nucleotide variants (SNVs). **c** Examples of *CYP2D6* copy number variations and their functional annotation. **d** Examples of *CYP2D7/2D6* hybrid genes. **e** Common tandem rearrangements in the *CYP2D* gene locus. The activity level boxes on the right are coded; red for a non-functional haplotype, orange for decreased activity, green for fully functional reference activity, and blue for increased activity.

The highly polymorphic *CYP2D6* gene has nine exons and is located on chromosome 22q13.2 neighbouring two paralogous pseudogenes, *CYP2D7* and *CYP2D8*, which further complicates genotyping^3,4^. To date, the Pharmacogene Variation (PharmVar) Consortium^5^ has catalogued over 130 different *CYP2D6* star alleles with varying levels of evidence (https://www.pharmvar.org, last accessed: 19-02-2020). The majority of *CYP2D6* star alleles are defined by specific combinations of single nucleotide polymorphisms (SNPs) and/or small insertions and deletions (indels) (collectively referred to as single/small nucleotide variants (SNVs) for the rest of this manuscript), which may either alter the function of the protein or be neutral. In addition, the *CYP2D6* gene locus contains a number of complex structural variants including full gene deletions, gene duplications and multiplications, as well as hybrid tandem rearrangements with the highly similar *CYP2D7*^6–8^. These allelic variants are graphically shown in Fig. 1.

The complexities in the *CYP2D* gene locus present a significant challenge in accurately genotyping *CYP2D6*. Currently, gold standard genotyping involves a battery of methods e.g. quantitative PCR analysis for CNV detection, allele specific XL-PCR, SMRT sequencing, and Sanger sequencing. However, these techniques are expensive, laborious and unscalable, especially for large cohort studies^9–11^. Further, although more economical, selectively targeting a subset of alleles through individually genotyping defining variants can have important clinical implications as inaccurate phenotype prediction is more likely to occur^11,12^. The widespread availability of next-generation sequencing (NGS) characterised by high-throughput parallel DNA sequence data generation provides a solution to the cost-limitations of experimental methods for genotyping *CYP2D6* and other pharmacogenes. The ever-decreasing cost of NGS facilitates whole-genome sequencing (WGS) in addition to providing options for the development of gene panels such as PGRNseq^13^.

In addition, NGS methods have the advantage of being unbiased, thus allowing for the detection of rare and novel variations, which are more likely to be deleterious^14^. Although there are important benefits associated with NGS-based genotyping, these approaches present computational challenges, mainly relating to the alignment of short-read data in regions of high sequence similarity. This makes variant calling and haplotyping genes such as *CYP2D6*, that are located in regions of high sequence similarity, challenging.

To address these challenges, bioinformatics tools that have recently been developed for calling star alleles in *CYP2D6* and other highly polymorphic pharmacogenes using genome sequencing data and/or targeted-capture panels such as PGRNseq, include Astrolabe (formerly Constellation)^15^, Aldy^16^, Stargazer^17^, VCF Annotator^18^, Cypiripi^19^, and PharmCAT^20^. These tools automate the detection of diplotype combinations based on PharmVar and the Pharmacogenomics Knowledgebase (PharmGKB) star allele catalogues thus facilitating clinical interpretation. The importance of these algorithms in *CYP2D6* phenotype prediction in clinical settings, and allele discovery in research studies, therefore cannot be overstated. However, to date, there is no comprehensive comparison of these tools. Astrolabe, Aldy, Stargazer, and PharmCAT are regularly maintained. However, PharmCAT does not perform star allele calling for *CYP2D6* directly, but rather uses Astrolabeś allele calling output for its unique clinical annotation step, which is based on current clinical implementation guidelines^20,21^.

We therefore provide a much-needed in-depth comparison of the performance of Astrolabe, Aldy, and Stargazer on a wide range of *CYP2D6* allelic variation using simulated data (entire *CYP2D* locus) and real data. The research participant-derived data used in the study are part of the Polaris pharmacogenomics cohort (https://github.com/Illumina/Polaris/wiki/HiSeqX-PGx-Cohort#Pratt2016) whose samples were originally collected as part of the HapMap and 1000 genomes projects^22^. The Centers for Disease Control and Prevention (CDC)-based Genetic Testing Reference Material Coordination Program (GeT-RM) has characterised these samples through extensive orthogonal testing by various laboratories^23,24^. In doing so, GeT-RM has contributed invaluable reference materials for benchmarking *CYP2D6* genotyping approaches.

In addition, we evaluate the impact of read depth on recall of *CYP2D6* CNVs for each tool. Given the differences in the allele calling approach of these algorithms, we further analyse the efficacy of an ensemble genotyping approach involving all three tools using the real data.

Through this comparative analysis, we provide important information on the strengths and limitations of current pharmacogene star allele calling algorithms. Our recommendations will serve as a valuable resource for the pharmacogenomics community regarding the use of these algorithms in clinical and research settings, and provide important data for developers aiming to improve the software or develop new ones. A summary of the main features of Astrolabe, Aldy, and Stargazer is given in Table 1.

**Table 1:** Properties of Astrolabe, Aldy, and Stargazer.

Tool	OS	Language	Central feature	NGS data	Input/ref	Output
Astrolabe	Linux	Java	Probabilistic	WGS	VCF	Diplotypes
	Mac		scoring system	PGRNseq^a^	BAM	Suballeles
					b37, b38	Phenotype
						All novel SNVs
Aldy	Linux	Python	Combinatorial	WGS	BAM	Diplotypes
	Mac		framework	PGRNseq	b37	Suballeles
						Putative novel
						core variants
Stargazer	Linux	Python	Statistical	WGS	VCF	Diplotypes
	Mac		phasing	PGRNseq	GDF	Phenotype
					b37	All novel SNVs

^a^Not yet optimised for Astrolabe’s CNV calling.

## Results

### Criteria

We evaluated Astrolabe, Aldy, and Stargazer using three criteria including;

- *Haplotype concordance*: The percentage of total haplotype calls that are consistent with the ground truth. The haplotype true positive rate is also known as the analytic specificity.
- *Diplotype (genotype) concordance*: The percentage of diplotype calls that are consistent with the ground truth (i.e. correctly calling both haplotypes for each sample).
- *Phenotype concordance*: Percentage of assigned phenotypes (based on activity scores) concordant to the reference materials.

The results reported here are specific to Astrolabe (v0.8.7.0), Aldy (v2.2.3), Stargazer (v1.0.7), and the datasets (simulated and real) used in this study. The goal was to have datasets with a wide variety of catalogued *CYP2D6* alleles. The simulated data (4618 test cases) do not represent actual population frequencies. In comparison, the GeT-RM WGS data (75 samples) are representative of African, Admixed American, European, and Asian allele distributions.

### Simulated datasets

Set 1 of our simulations comprised all theoretically possible diplotype combinations (homozygous and heterozygous) derived from PharmVar-catalogued *CYP2D6* SNV-defined haplotypes. Set 2 comprised diplotypes with at least one structural variant (*CYP2D6* gene deletion (*5), duplications, exon conversions and hybrid rearrangements i.e. *CYP2D6/2D7* and *CYP2D7/2D6* hybrids). These structural variations are comprehensively described by PharmVar (https://www.pharmvar.org/gene-support/Variation_CYP2D6.pdf). For a complete list of all the *CYP2D6* haplotypes used for generating simulations in this study, see Supplementary Data Set 1.

### Performance based on simulated data

In set 1 (SNV-defined alleles), the concordance of Astrolabe and Aldy for homozygous diplotypes was comparable as they correctly identified 142 (92%) and 148 (96%) diplotypes, respectively, out of 154 diplotypes (Table 2, Run 1). However, Stargazer had lower concordance (135 i.e. 88%) for the homozygous diplotypes. On further investigation, we found 18 cases where Stargazer prioritised reporting background functionally annotated alleles as the main result for haplotypes that have uncertain function. For example, Stargazer reported *58/*58 as *17/*17 given that *58(unknown function) and *17(decreased function) both have the T107I variant. In such cases, Stargazer reported the expected allele(s) ambiguously with other candidate solutions (see Table 2, Supplementary Data Set 2, and Supplementary Information for additional details).

**Table 2:** Concordance of Astrolabe, Aldy, and Stargazer on test cases (*N* = 154) homozygous for SNV-defined star alleles at 30×.

	Astrolabe		Aldy		Stargazer
	Run 1	Run 2	Run 1	Run 2	
Match	142 (92%)	152 (99%)	148 (96%)	149 (97%)	135 (88%)
Mismatch	9 (6%)	1 (1%)	4 (3%)	3 (2%)	19 (12%)
Ungenotyped	3 (2%)	1 (1%)	2 (1%)	1 (1%)	0

Inconsistencies			
Algorithm	Challenges	Discordant cases	Notes
Astrolabe	Undefined suballeles	*2.014, *4.002, *4.003, *4.007, *4.009, *52.002, *56.003	Ambiguous calls or miscalls
	Indel-defined alleles	*18.001	Position discordance with database
Aldy	Undefined suballeles	*12.002, *71.003	Ambiguous calls or miscalls
	Indel-defined alleles	*18.001, *20.001, *38.001	Position discordance with database
Stargazer	Indel-defined alleles	*20.001	Position discordance with database
	Alleles with unknown or uncertain function and/or rare alleles	18 alleles including *30.001, *37.001, *58.001, *52.001, *64.001, *65.001, and *73.001 (Supplementary Data Set 2)	Stargazer reports background alleles as the main haplotypes in these cases. Exact haplotypes (matching truthset) in the sample(s) are reported among other candidate haplotypes. (See discussion for details)

The complete list of genotypes called per sample is provided in Supplementary Data Set 2.

Run 1 and Run 2 indicate results before and after defining missing suballeles in the allele tables, respectively. There was only one run for Stargazer as it does not typically call suballeles.

Calling some indel-defined alleles was challenging for all the tools due to left/right shifts in the alignments. These shifts emphasise challenges of using short-read NGS data for genotyping *CYP2D6* as they caused position discordance in the input data (BAM and/or VCF) and the algorithms’ definitions (see Table 2 for examples of problematic indel-defined alleles). Discordant cases for Astrolabe and Aldy involved undefined suballeles as well (Table 2), resulting in ambiguous genotypes or miscalls. The concordance of Astrolabe and Aldy increased to 99% and 97%, respectively, after updating their allele databases with previously undefined suballeles (Table 2, Run 2).

**Fig. 2:**
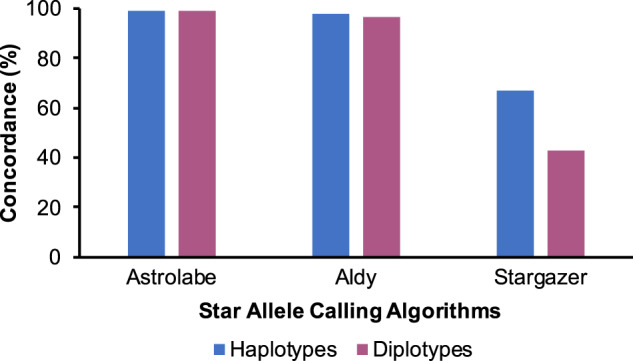
Concordance of Astrolabe, Aldy, and Stargazer for all theoretically possible *CYP2D6* diplotype combinations (homozygous and heterozygous) comprising SNV-defined haplotypes with PharmVar definitive or moderate level of evidence from our starting set. We left the allele databases of the three tools as is in order to examine the effect of the undefined suballeles on diplotype calling. The sequencing coverage for each test case was 30× and default parameters were used for each tool.

We next considered tool concordance on all theoretically possible diplotype combinations of *CYP2D6* SNV-defined haplotypes with definitive or moderate PharmVar level of evidence (*N* = 4560 i.e. 95 choose 2 plus 95). The main diplotypes called by all three tools are shown in Supplementary Data Set 3. The concordance of Astrolabe and Aldy was again comparable (99% and 97%, respectively), whereas Stargazer was concordant for only (43%) of the possible diplotypes (Fig. 2). Stargazer’s lower recall can be attributed to not only the aforementioned differences in reporting uncertain function alleles but also to the limitations of the statistical phasing process central to the Stargazer pipeline. For example, the *74/*48 combination involving a rare African-specific haplotype (*74) and a rare East Asian-specific haplotypes (*48) was called as *1/*1 by Stargazer. Homozygous cases of these alleles are correctly called by Stargazer via manual phasing. However, rare heterozygous combinations are more difficult to call since population structure affects statistical phasing. Notably, some alleles are correctly called using Stargazer’s “phasing by haplotype extension algorithm” that complements the statistical phasing (https://stargazer.gs.washington.edu/stargazerweb/). Astrolabe and Aldy do not do statistical phasing; hence, they are virtually not affected by population structure.

For all three tools, the concordance for haplotypes was much higher than that for diplotypes. This underscores the challenge of consistently calling both haplotypes for each sample in a highly polymorphic gene such as *CYP2D6*. We show the true extent of this later on with the real data that follows true population distributions.

All three tools accurately genotyped simulated samples with at least one *CYP2D6* full gene deletion allele (*5) (i.e. CN = 0,1) as indicated in Table 3. The notable discordances were samples that contained a duplicated allele on the second haplotype (e.g. *5/*29×2 was called as *29/*29). Astrolabe in particular also had challenges calling the *5/*5 diplotype (CN = 0). Astrolabe reported failed BAM quality control for the *5/*5 test cases. Running the algorithm by skipping quality control unsurprisingly yielded haplotype calls matching GRCh37 (i.e. *2M/*2M, indicating absence of non-reference SNVs). Varying the coverage from 30× to 100× had very little effect on the CYP2D6*5 recall of Aldy and Stargazer. However, Astrolabe detected 5 from 20 CYP2D6*5 alleles at 60× compared to 10 from 20 at 30× and 100×. The miscalls were observed mainly in samples with CN = 1.

**Table 3:** Summary of correctly called structural variations by Astrolabe, Aldy, and Stargazer in set 2.

Set 2	Truth	Astrolabe			Aldy			Stargazer		
		30×	60×	100×	30×	60×	100×	30×	60×	100×
Full gene deletion (*5)	20	10	5	10	15	14	15	15	15	15
Copy number gain^a^	52	29	25	25	52	50	52	47	45	45
Resolved duplicated/ multiplicated alleles	61	4^c^	4^c^	4^c^	53	49	51	54	51	48
Hybrids^b^	107	3	8	8	72	66	67	31	30	28
Non-hybrid tandem	4	0	0	0	4	4	4	0	0	0
Ungenotyped (defaults)		0	0	0	1	3	2	18	16	19

The complete list of genotypes called per sample is provided in Supplementary Data Set 4.

^a^Due to gene duplications/multiplications.

^b^Collectively representing exon conversions and gene hybrids involving *CYP2D6* and *CYP2D7*.

^c^Determined only for homozygous allele cases.

For copy number gains (CN > 2), Aldy and Stargazer outperformed Astrolabe by detecting up to 50% more duplication/multiplication events. By default, Aldy and Stargazer resolve copy number gains further to report which of the star alleles was duplicated or multiplicated. However, for our simulated test cases with high copy number, some discordant tandem arrangements were reported by Aldy and Stargazer. For example, Aldy genotyped the test case *1×4/*2×8 as *1×3+*63/*2×7+*79, whereas Stargazer genotyped it as *1×6/*34×6. Genotypes with such high *CYP2D6* copy number are rare but possible as shown previously^25^. It is worth mentioning that Aldy was the only algorithm that resolved the *90+*1 non-hybrid tandem arrangements.

In contrast to Stargazer and Aldy, Astrolabe does not actively resolve duplicated/multiplicated alleles but rather generically indicates that a gene duplication has been detected. Similarly by default, Astrolabe does not distinguish between a duplication and a multiplication but rather generically indicates the presence of a gene duplication if copy number gain is detected. The concordance for duplications/multiplications was more or less equally good at 30×, 60× and 100× for Aldy and Stargazer (Table 3). For Astrolabe, there was no evidence to suggest that increasing coverage from 30× to 60× or 100× increases recall of duplications. This could be due to limitations of using short-read NGS data for interrogating the complex *CYP2D6* region.

Regarding gene exon conversions and hybrid rearrangements, Aldy consistently called more of such alleles at 30× (72 out of 107) compared to Stargazer (31 out of 107) and Astrolabe (3 out of 107). Astrolabe consistently called *82 at 30×, 60×, and 100× unlike Aldy and Stargazer, and then consistently called the *68+*4 tandem only at 60× and 100×. As observed for duplication events, increase in coverage from 30× to 60× and 100× did not considerably affect Aldy and Stargazer’s performance for calling hybrids (see Table 3 and Supplementary Data Set 4 for additional details).

### Comparison of *CYP2D6* star allele calling performance on real data

To evaluate the genotyping accuracy of Aldy, Astrolabe, and Stargazer on real data, we applied all three algorithms to 75 ethnically diverse samples from the GeT-RM project. GeT-RM previously characterised 137 DNA samples from Coriell cell lines for 28 genes, including *CYP2D6*, through targeted genotyping by selected laboratories^23^. GeT-RM has recently re-characterised these 137 samples and genotyped 42 new samples to ascertain complex rearrangements and rare variants in *CYP2D6*^24^. Of the combined 179 samples, 75 currently have publicly available high coverage WGS data (see Data availability section for details). We used published consensus *CYP2D6* genotypes^23,24^ for these samples as the Gold Standard calls except for sample NA18519. GeT-RM used only panel-based genotyping for this sample resulting in the *1/*29 diplotype call. However, the *106 defining variant 3878G>A as well as the *29 defining variants were detected from NGS. Therefore, *106/*29 is considered to be the true positive call for sample NA18519 for this analysis.

**Fig. 3:**
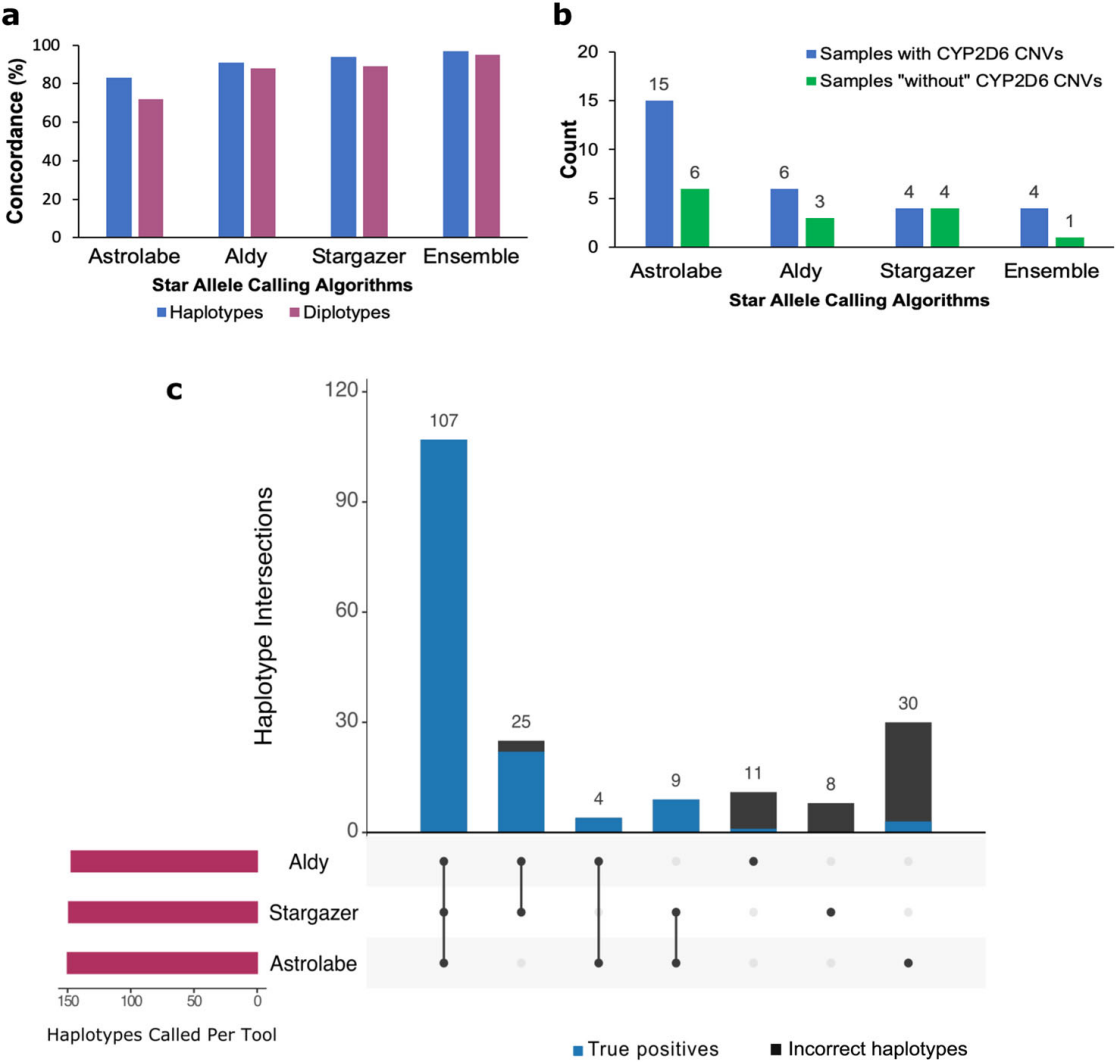
Performance of Astrolabe, Aldy, and Stargazer on 75 WGS GeT-RM samples. **a** Concordance of each algorithm to the GeT-RM consensus calls. **b** Cases with discordant genotypes. The green colour represents samples “without” *CYP2D6* CNVs (most of the samples may have the intron 1 conversion), whereas the blue colour represents samples with allele-defining *CYP2D6* CNVs. **c** Overlap of haplotypes called by each algorithm. As shown ensemble call sets have high concordance. Haplotypes called by one tool but not confirmed with any of the others have low true positive rate.

When applied to the GeT-RM WGS samples, both Astrolabe and Stargazer assigned diplotypes for 75 of 75 samples. One sample could not be genotyped by Aldy with default parameters. Stargazer had the highest genotype concordance (89%—67 of 75 diplotypes) to the ground truth followed by Aldy (88%—66 of 75 diplotypes) and then Astrolabe (72%—54 of 75 diplotypes). The haplotype concordance followed a similar trend i.e. 94% (141 of 150 haplotypes), 91% (136 of 150 haplotypes), and 83% (125 of 150 haplotypes) for Stargazer, Aldy, and Astrolabe, respectively (Fig. 3a).

Notably, the ensemble genotyping approach (2 from 3 rule) had the highest diplotype concordance (95%—71 of 75 diplotypes) and haplotype concordance (97%—145 of 150 haplotypes).

Samples with *CYP2D6* CNVs were more challenging to call for all the algorithms notably Astrolabe (15 of 75 samples) (Fig. 3b). Astrolabe particularly had challenges calling *36, a *CYP2D6*/2D7 hybrid gene that frequently occurs singly or in tandem with *10. Tandem rearrangements involving *36 present in the GeT-RM dataset include *36×2, *36+*10, and *36×2+*10. Aldy and Stargazer consistently detected the more common *36+*10 arrangement but could not call all copies correctly for the more complex *36×2 and *36×2+*10 arrangements (see Supplementary Data Set 5 for detailed genotype calls).

All the three tools consistently called the *68+*4 tandem arrangement except for one case where Astrolabe called sample NA21781 as *2/*4 (possible duplication) instead of *2×2/*68+*4. *68 is a non-functional *CYP2D6*/2D7 hybrid gene with an intron 1 breakpoint. The *68+*4 haplotype is therefore non-functional as *4 is also a non-functional allele.

As shown in Fig. 3c, haplotype calls by one tool that are not confirmed by either of the other two tools are mostly incorrect. The caller overlap between Aldy and Stargazer was more prominent than either tool’s intersection with Astrolabe because of Astrolabe’s lower recall for structural variations. The ensemble genotyping approach reduced the number of discordant genotype calls especially for samples “without” *CYP2D6* CNVs (many of these have haplotypes with the neutral intron 1 conversion). However, some alleles defined by *CYP2D6* CNVs (especially aforementioned *36 hybrid arrangements) proved to be challenging even with ensemble calling (Fig. 3b). In addition, the ensemble genotyping approach had two ambiguous calls (from samples NA19908 and NA18540) arising from discrepant calls from all three tools (see Supplementary Data Set 5).

### Comparison of *CYP2D6* phenotype prediction concordance

**Fig. 4:**
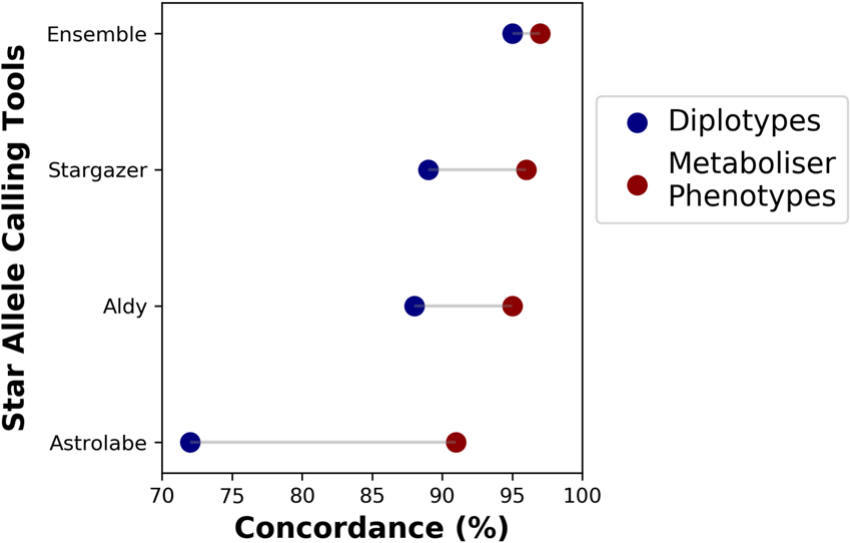
Comparison between the CYP2D6 diplotype concordance and the phenotype prediction concordance for each algorithm based on 75 GeT-RM samples and the Activity Score system. All three algorithms and the ensemble approach have relatively high phenotype concordance thus underscoring their clinical utility even for some cases with inconsistent diplotype calls.

We next evaluated the clinical accuracy of Astrolabe, Aldy, Stargazer, and the ensemble approach. We followed the current consensus clinical implementation guidelines^21,26^ to assign activity scores corresponding to the genotype calls and compared them to the GeT-RM consensus reference. All the three algorithms had much higher phenotype concordances compared to their diplotype concordances (Fig. 4). This is due to the fact that even though some samples may not be accurately genotyped, the activity score or phenotype group of the reported diplotype could be the same as that of the truth call. For instance, sample NA18565 was called as *10/*36+*10 (Stargazer and Aldy) and *10/*10 (Astrolabe) instead of *10/*36×2 (truth call). All these genotypes denote an intermediate metaboliser phenotype. Aldy (95%) and Stargazer (96%) had comparably higher phenotype concordance than Astrolabe (91%) because they were able to consistently genotype more samples with *CYP2D6* CNVs as mentioned earlier. Notably, the ensemble approach had the highest phenotype concordance (97%) and it was comparable to its diplotype concordance (95%). In some cases, we obtained phenotype “no calls” due to ungenotyped samples e.g. for Aldy(1) and the ensemble approach (two ambiguous cases).

## Discussion

Accurately calling star alleles in *CYP2D6* is crucial to inferring phenotype from genotype. However, *CYP2D6* is one of the most challenging genes to genotype largely because of the complexity of its genomic locus. The limitations of using short-read NGS data compound the problem even further. Herein, we have benchmarked three algorithms that use largely different approaches to call *CYP2D6* star alleles from high-throughput sequence data.

We used simulations to test the performance of each algorithm on a wide variety of *CYP2D6* allelic variation i.e. 154 SNV-defined haplotypes (many have the *CYP2D7* intron 1 conversion), and over 50 different structural variant arrangements (Supplementary Data Set 1). We also assessed the accuracy of each algorithm using real WGS data from CDC-based GeT-RM project.

Overall, Astrolabe and Aldy call more combinations of SNV-defined *CYP2D6* star alleles (homozygous and heterozygous) compared to Stargazer. Stargazer faces a challenge in calling rare (frequency < 0.1%) SNV-defined alleles due to its reliance on statistical phasing. Although Stargazer has supplementary algorithms to phase rare variants that are not present in the reference panel, we found that they work best in homozygous cases but have lower accuracy in heterozygous cases. Stargazer also had discordances due to its difference in reporting alleles with unknown/uncertain function. For such alleles, Stargazer currently prioritises to report background alleles for which the function is known as the main haplotypes. However, it is difficult to infer the exact alleles in the sample as they are reported in a separate column ambiguously with other candidate alleles. Notably, homozygous cases of these alleles are assigned unfounded phenotypes. For example, *58/*58 (unknown metaboliser status) is called as *17/*17 (intermediate metaboliser) by Stargazer, whereas *58 is reported among candidate alleles for haplotype 1 and 2.

Notably all three algorithms depend on the comprehensive definition of alleles (and suballeles especially for Astrolabe and Aldy) to avoid ambiguous calls and miscalls. Indel-defined star alleles are a major challenge for all three algorithms especially where the core SNV occurred in a repeat region causing the aligner to pick the left-most possible position in the alignment (BAM) file. Core indels for *18, *20, *38, *40, and *42 all appear in unexpected positions due to this problem. Miscalling these alleles could have potential clinical implications. Even though the three algorithms have corrected for some of these changes in their allele definitions, the complexity of their locus can affect coverage and variant quality scores. Of note, other alleles with indel core variants such as *3, *6, *9, *19, and *21 were consistently called.

Regarding the calling of *CYP2D6* structural variants, duplications/multiplications and deletions were considerably easier to call compared to hybrid rearrangements. Aldy had the highest concordance followed by Stargazer, and then Astrolabe. Notably, Astrolabe does not distinguish between duplications and multiplications (i.e. identifies all scenarios as duplications), and also does not resolve the exact duplicated gene copy (or copies) for samples where it detects copy number gains. This could affect phenotype prediction depending on the diplotype combination in a sample. Astrolabe also had challenges detecting the *5/*5 diplotype (CN = 0) probably because its current model for structural variant calling was not trained on enough cases with zero copy number. In addition, for hybrid rearrangements, Astrolabe consistently detected only *82 (has an exon 2 conversion) and *68+*4 (at 60× and 100×). As with the duplication events, the effect of Astrolabe’s inconsistency in calling hybrid rearrangements on phenotype prediction depends on the entire diplotype combination of an individual.

Stargazer had the intermediate performance for calling structural variants of the three tools. It was able to phase CNVs and detect more hybrid rearrangements compared to Astrolabe while also producing visual plots (Supplementary Fig. 1) of the structural variants in the process. However, Stargazer had the highest number of indeterminate (i.e. ungenotyped) diplotypes for samples with structural variants. Notably, Stargazer still produced copy number and allele fraction plots for these samples. However, ungenotyped calls can be a major hindrance to automated phenotype prediction in practice.

Aldy performed best in calling *CYP2D6* structural variants. However, as with Astrolabe and Stargazer, it had challenges in reconstructing complex hybrid rearrangements such as *79+*68+*4 and *79+*68+*4×2, and it also incorrectly indicated the presence of hybrids such as *63 and *79 for samples with high copy number. Although high copy number diplotypes such as *1×4/*2×8 are rare, it is important to examine whether they can be picked up by these algorithms. This is particularly important given that some populations are largely understudied and may have individuals with such alleles.

To evaluate the performance of Astolabe, Aldy, and Stargazer on real data, we used 75 sequences from the 1000 Genomes Project for Coriell reference samples that have been re-characterised through the GeT-RM project. Astrolabe and Stargazer were able to call diplotypes for 100% of the samples, whereas one sample could not be diplotyped using Aldy’s default parameters. However, the concordance of Aldy and Stargazer was comparably higher than that for Astrolabe mainly due to their superiority in calling *CYP2D6* CNVs. Although the 75 GeT-RM WGS samples used for this analysis depicted the complexity and diversity in *CYP2D6* pharmacogenomic variation, they did not include a number of rare alleles. Future availability of WGS data for the remaining 104 GeT-RM reference materials will provide the opportunity to test these algorithms against a more comprehensive distribution of *CYP2D6* star alleles.

As with the simulated data, gene deletions as well as duplications/multiplications were more consistently called compared to hybrid rearrangements. However, Astrolabe missed more deletions and duplications than Aldy and Stargazer did. This could be attributed to Aldy and Stargazer’s coverage normalisation strategies during structural variant detection that are not utilised by Astrolabe.

Regarding hybrids, the *68+*4 non-functional tandem arrangement was consistently called by all three algorithms. Notably, Astrolabe could not differentiate *36 from *10. On the other hand, Aldy and Stargazer consistently called the *36+*10 arrangement but could not differentiate more complex arrangements such as *36×2, *36×2+*10. This can be attributed to incomprehensive definition of these haplotypes within the algorithms and also the limitations of using short-read NGS data.

We also assessed the overlap of Astrolabe, Aldy, and Stargazer calls as well as the practical application of ensemble *CYP2D6* star allele calling. Simple ensemble calling could be useful in obtaining high-confidence *CYP2D6* star allele calls both in the clinical setting and in large-scale population studies. Through obtaining high-confidence star allele calls, the number of samples requiring experimental validation (e.g. those found to have putative novel alleles and structural variants) would be reduced to a smaller subset of the study population. We generated the ensemble call set by taking a consensus of Aldy, Astrolabe and Stargazer allele calls for each sample on a 2 from 3 rule. We obtained 100% concordance for haplotypes (107) that were called by all three algorithms, and only 5 discordant genotypes compared to Astrolabe (21), Aldy (9), and Stargazer (8) from 75 samples. Based on the challenges and limitations of each algorithm and of using short-read NGS data, we recommend the use of all three algorithms to perform highly accurate ensemble calling. The ensemble calling is also practical as the typical runtime for each of three algorithms from our workflows was less than 1 min per sample while using 4 CPU cores.

Although it is quite straightforward to obtain high-confidence SNV-defined star allele calls using ensemble calling, it is more challenging to get unambiguous high-confidence diplotype calls for individuals with complex structural variations especially the hybrid rearrangements. This follows from the differences in accuracy of the three algorithms for different forms of *CYP2D6* allelic variation described above. A worry with ensemble calling in the clinic would be how to resolve the discrepancies among the tools. These samples would require orthogonal testing to resolve the number and exact sequence content of their *CYP2D6* copies. Further work is required to build an automated ensemble allele calling and phenotype prediction pipeline. This will heavily depend on achieving uniformity in the precision of reporting star alleles and suballeles by all the algorithms in their future updates.

All three algorithms had much higher phenotype concordance compared to their diplotype concordance implying that their clinical accuracy does not necessarily match their genotyping accuracy. Notably, the ensemble genotyping approach had comparably high phenotype and diplotype concordance in contrast to Astrolabe, Aldy, and Stargazer. This implies that ensemble genotyping followed by phenotyping, minimises incorrect predictions of individual *CYP2D6* diplotypes as well as metaboliser status for which the three algorithms could be prone if used singly.

The pros and cons of Astrolabe, Aldy, Stargazer, and the ensemble approach based on our analysis are summarised in Table 4.

**Table 4:** Pros and cons of current *CYP2D6* genotyping algorithms.

Algorithm	Advantages	Disadvantages
Astrolabe	High accuracy for calling catalogued SNV-defined alleles.	Lower recall for *CYP2D6* CNVs and hybrid rearrangements compared to Aldy and Stargazer
	Supports hg19 and hg38.	Prone to ambiguous calls and miscalls if alleles/suballeles are not comprehensively defined
	Performs variant calling error correction.	Does not discriminate duplicated alleles in a sample with copy number gain
		(generically reports presence of a duplication)
	Easy to run (one-liner command).	Does not distinguish between duplication events and multiplication events
	Performs automated phenotype prediction.	
Aldy	High accuracy for calling catalogued SNV-defined alleles.	Aldy supports only hg19 as of v2.2.3
	Highest accuracy of the three tools in calling *CYP2D6* structural variations.	Prone to ambiguous calls and miscalls if alleles/suballeles are not comprehensively defined
	Easiest to run of the three tools as it requires only the BAM file on any system (even a normal laptop).	Prone to some erroneous calls when using default arguments due to sequencing noise
Stargazer	Supports various file inputs and imputation.	Low recall for rare alleles especially in heterozygous combinations
	Supports user-defined references for phasing and/or phased VCF input.	Affected by linkage disequilibrium as it has to do statistical reference-based phasing
	Provides tools for viewing metrics of key variants in a sample.	Prone to “no calls” for complex hybrid arrangements/combinations
	Outputs coverage plots for visually examining change-points for samples with CNVs.	Stargazer v1.0.7 supports only hg19
	Performs automated phenotype prediction.	Does not call suballeles as of v1.0.7
	Easy to run (one-liner command).	Has inconsistent reporting of alleles with uncertain function (reports background functionally annotated alleles for these cases as of v1.0.7)
Ensemble	Comparably high diplotype/haplotype concordance.	Difficult to resolve complete discrepancies between the genotypes of the three tools
	Resolves single tool deficiencies.	Prone to ambiguous calls especially for structural variations
		No automated pipeline as of now as the three algorithms are being updated on a regular basis and the reporting of alleles/suballeles is non-uniform

Our approach had some limitations. Firstly, we used simulated data for most of the benchmarking especially for rare alleles and structural variants. Therefore, we did not capture the alterations in performance caused by some of the features present in real data for these cases. However, publicly available reference datasets with validated *CYP2D6* diplotype calls are limited and they also have drawbacks as they do not cover a wide variety of *CYP2D6* allelic variation. The GeT-RM reference materials are more or less comprehensive; however, public WGS data is not yet available for all the samples or biobank scale cohorts. The different relative performances between the algorithms on simulated data and real data are attributed to the different distribution of alleles (i.e. fewer rare alleles in the real data). Our exhaustive simulation enabled us to test the genotyping algorithms on a wider array of *CYP2D6* variations. On the other hand, the small cohort of real world patients was very helpful for evaluating results from the algorithms against those obtained by using orthogonal techniques. The 75 GeT-RM samples are representative of 24 *CYP2D6* major star alleles (17 SNV-defined haplotypes plus 7 structural variants) compared to the 204 haplotypes (154 SNV-defined haplotypes plus 50 structural variants) represented in our simulated data.

We also did not compare the performance of the three tools across simulated or real NGS data from targeted custom-capture panels such as PGRNseq. These panels greatly reduce the cost of sequencing and enhance genotyping turnaround time. Further, we did not compare the influence of the aligner, BWA-MEM versus other aligners such as GSNAP^27^ and NovoAlign (http://www.novocraft.com).

It is important to note that these algorithms face a major challenge of genotyping individuals with novel SNVs which may either be neutral or allele-defining (based on unpublished data). We did not test for this in this study as the reference materials used did not have novel star alleles. Ideally, for such cases, the two background star alleles would be called but the novel SNV(s) would be ambiguously assigned to either haplotype. Experimental validation using approaches such as Sanger sequencing, XL-PCR and/or long-read sequencing (e.g. PacBio, and Oxford Nanopore) would then be required to unequivocally determine the phase of these novel SNVs. Caution also needs to be taken in cases of complex and/or novel CNVs as they might be misreported by the algorithms. Nonetheless, combining short-read NGS (especially WGS) and tools such as Astrolabe, Aldy, and Stargazer (or their ensemble) is crucial to characterisation of pharmacogene variation in a cost-effective manner by reducing the number of cases that require the more expensive experimental genotyping approaches. Lastly, even though our comparison is focused on *CYP2D6*, we acknowledge that Astrolabe, Aldy, Stargazer, and PharmCAT (not included in the analysis) are extremely useful in genotyping other pharmacogenes for example *CYP2C9*, *CYP2C19*, *CYP2A6*, *CYP2B6*, *CYP3A4*, and *CYP3A5* as shown previously by others^15,16,20,28^ (see also Supplementary Data Set 6).

Through this benchmarking study, we have examined the major differences as well as strengths and limitations of three algorithms that perform the challenging task of genotyping *CYP2D6* and other highly polymorphic pharmacogenes using short-read NGS data. Some of the inaccuracies reported were due to sequencing noise in the simulated and real data. This supports the notion that clinical-grade NGS data (preferably high coverage WGS) is required for effective use of these algorithms in the clinical setting. It is also clear that more *CYP2D6* pharmacogenetic studies especially in understudied populations would add to the completeness of the allele catalogue in PharmVar and in the allele definitions of these in silico genotyping algorithms hence enhancing their utility across populations.

## Methods

### Ethics

Ethical approval was not required for this study as we used simulated and publicly available WGS data only.

### Brief description for each algorithm

Detailed descriptions of all three algorithms are given in their original publications^15–17^.

Briefly, the Astrolabe (v0.8.7.0) algorithm uses probability scoring to identify the most likely *CYP2D6* diplotype match based on variant positions and zygosity from input variant call format^29^ (VCF) or gVCF files. Astrolabe also corrects for variant calling errors by using default or user-derived sensitivity and specificity of the variant calling approach. In addition, for WGS data, Astrolabe uses BAM^30^ files for CNV calling by comparing the alignment depth of coverage ratios of the *CYP2D6* exonic regions to the coverage across various control regions along chromosome 22. Astrolabeś output includes *CYP2D6* diplotypes for each sample, special notes on structural variants detected, suballeles, and diplotype activity information.

Aldy (v2.2.3) differs from Astrolabe by using a combinatorial approach to reconstruct the sequence content in each copy of a target polymorphic gene based on data from a sample’s SAM^31^, BAM or CRAM^32^ file. Aldy leverages integer programming solvers such as Gurobi^33^, SCIP^34^, and CBC^35^ for timely solution of problem instances (copy number estimation, major and minor star allele prediction) that would otherwise take an extended period of time to solve. In addition, Aldy performs a coverage normalisation step, which corrects for errors when calling alleles and CNVs from non-uniform coverage sequence data generated from target panels such as PGRNseq.

The Stargazer (v1.0.7) pipeline retrieves variant information from VCF files generated using GATK-HaplotypeCaller^36^, then performs statistical haplotype phasing using Beagle^37^, and thereafter assigns *CYP2D6* diplotypes based on haplotype matches with a reference star allele table. The Stargazer pipeline also includes supplementary algorithms for phasing variants that are not present in the reference VCF (default in v1.0.7 is the 1000 genomes reference panel). Of note, Stargazer also accepts phased VCF files, chip-generated VCFs, and provides the option for imputation. In addition, for structural variant detection, Stargazer requires read depth information for the *CYP2D6* and *CYP2D7* regions as well as a control gene region (e.g. *EGFR*, *RYR1*, and *VDR*), generated using GATK-DepthOfCoverage^36^. The main output of Stargazer includes *CYP2D6* genotype data for each sample, interactive visual plots for CNVs, and predicted phenotype assignment based on the activity score system^38,39^. In case a user has an automation platform installed, such as the Sun Grid Engine (http://gridscheduler.sourceforge.net/htmlman/manuals.html), Stargazer can be run using the BAM file as input.

### Simulation of benchmark datasets

In order to compare the performance of Aldy, Astrolabe, and Stargazer in calling a large variety of *CYP2D6* haplotypes, we created simulated datasets with known *CYP2D6* diplotypes for unrelated individuals, following the approach described by Numanagić et al.^19^ with some modifications.

Maternal and paternal *CYP2D* locus sequences with respect to the human reference genome, GRCh37 (Chr22:42518000–42555000) were constructed separately for the simulation process. For cases with no allele-defining structural variants, GATK FastaAlternateReferenceMaker^36^ was used to mutate the *CYP2D6* portion of the maternal and paternal *CYP2D* sequences by giving *CYP2D6* haplotype VCF files from PharmVar (version 4.0.4) as input. Haplotype sequences with *CYP2D6/2D7* intron and/or exon conversions were constructed in a similar way since PharmVar gives details of all the SNVs arising from these structural changes in the VCF files.

*CYP2D6* gene deletions were generated by creating breakpoints within the REP6 and REP7 regions as described by Gaedigk et al.^40^ and Steen et al.^41^ previously. *CYP2D6* duplications were generated by concatenating *CYP2D6* sequences containing desired mutations. For haplotypes with more complex structural variations such as CYP2D6 *61, CYP2D6*63, and subvariants of CYP2D6 *13, we replaced reference *CYP2D6* and/or *CYP2D7* portions of the *CYP2D* locus with haplotype sequences deposited in the NCBI Nucleotide database by Kramer et al.^6^, Black et al.^8^, and Gaedigk et al.^7^.

We simulated 101-bp paired-end reads (Illumina Hiseq2000) corresponding to maternal and paternal *CYP2D* haplotype sequences of each sample using the simNGS software (https://www.ebi.ac.uk/goldman-srv/simNGS/). simNGS replicates NGS-like noise intensity, base quality, and read error distributions by using runfiles trained from real sequencing experiments.

In order to account for the structural variant detection approaches used by Astrolabe and Stargazer, we simulated reads corresponding to CNV-neutral regions used by the algorithms and added them to the FASTQ files for each sample. The GRCh37 sequences (Chr22:19882000–19908000 and Chr22:44218000–44233000) contain control regions used by Astrolabe. For Stargazer, we used the *RYR1* gene locus (Chr19:38924340–39078204) as the control region. The CNV-neutral region that Aldy uses (Chr22:42547463–42548249) is covered by our simulation of the whole *CYP2D* locus.

Furthermore, to assess how various sequencing coverage affected Aldy, Astrolabe, and Stargazerś *CYP2D6* structural variant calling, we simulated read data with different coverage profiles that are frequently implemented in research and clinical settings (~30×, 60×, and 100×).

In total, we generated 4618 simulations for set 1, comprising only SNV-defined alleles. For set 2, where each sample had at least one haplotype with allele-defining *CYP2D6* strucural variant(s), we had 3 replicates of 121 samples at different coverages as mentioned above. We excluded some *CYP2D6* star alleles from our analysis, including *32, *99, *110–*139, either because they were not defined by all the three tools or they were still under curation by PharmVar. However, undefined suballeles were included to check for their effect on the tools’ performance.

### Real data

Regarding the real data, we used 75 samples from the CDC’s GeT-RM project for which WGS data is publicly available. These samples were originally collected as part of the 1000 genomes and HapMap projects and were sequenced on Illumina HiseqX platform (2 × 150 bp reads) (https://github.com/Illumina/Polaris/wiki/HiSeqX-PGx-Cohort#Pratt2016).

### Variant calling

We aligned all our simulated read sets against the human reference genome (build 37) using BWA-MEM^30^. We used the b37 reference in our analysis because it is supported by all three algorithms (Table 1). The GeT-RM WGS data were already in BAM format (b37) on retrieval. We obtained gVCF files from BAM files using GATK-HaplotypeCaller followed by generation of VCF files with GATK GenomicsDB and GenotypeGVCFs^36^.

### *CYP2D6* star allele calling

We performed *CYP2D6* star allele calling separately for Aldy, Astrolabe and Stargazer using default parameters, as well as parameters recommended by the authors in cases of debugging and CNV calling. BAM files were used as input for Aldy, whereas Astrolabe required both BAM and VCF files. For Stargazer, we first generated depth of coverage (GDF) files, required for calling structural variants, using GATK3 DepthofCoverage. The GDF files were then used as input together with VCF files for each run. We included reference samples without structural variants to account for Stargazer’s intersample normalisation.

For the GeT-RM samples, we determined consensus genotypes (ensemble) from the separate genotype calls of Astrolabe, Aldy and Stargazer using the 2 from 3 rule. Truth calls for these samples were obtained from previous publications by others^23,24^.

### Reporting summary

Further information on research design is available in the Nature Research Reporting Summary linked to this article.

## Supplementary information

Supplementary Information

Supplementary Data Set 1

Supplementary Data Set 2

Supplementary Data Set 3

Supplementary Data Set 4

Supplementary Data Set 5

Supplementary Data Set 6

Reporting Summary

## Data Availability

The real datasets used in this study are publicly available from the European Nucleotide Archive (https://www.ebi.ac.uk/ena/data/view/PRJEB19931). Samples NA12878, NA12891, and N12892 can also be downloaded from the 1000 genomes project repository (https://www.internationalgenome.org). Metadata for all samples can be found at https://github.com/Illumina/Polaris/wiki/HiSeqX-PGx-Cohort#Pratt2016. The VCF files used to create hg19 consensus haplotype sequences for simulations are publicly available from the PharmVar database (https://www.pharmvar.org/gene/CYP2D6). All the fasta sequences used to generate *CYP2D6* CNV simulations are available at https://github.com/twesigomwedavid/CYP2D6-gt-algorithms.

## Code availability

The code used for this analysis is publicly available at https://github.com/twesigomwedavid/CYP2D6-gt-algorithms.
