# Supplementary material for: A systematic comparison of pharmacogene star allele calling bioinformatics algorithms: a focus on CYP2D6 genotyping
Source: NPJ Genom Med. 2020 Aug 3;5:30. doi: 10.1038/s41525-020-0135-2 (PMC7398905; doi:10.1038/s41525-020-0135-2)
Supplement: Supplementary file 1 — Supplementary Information [file 41525_2020_135_MOESM1_ESM.pdf]

Supplementary Figure 1

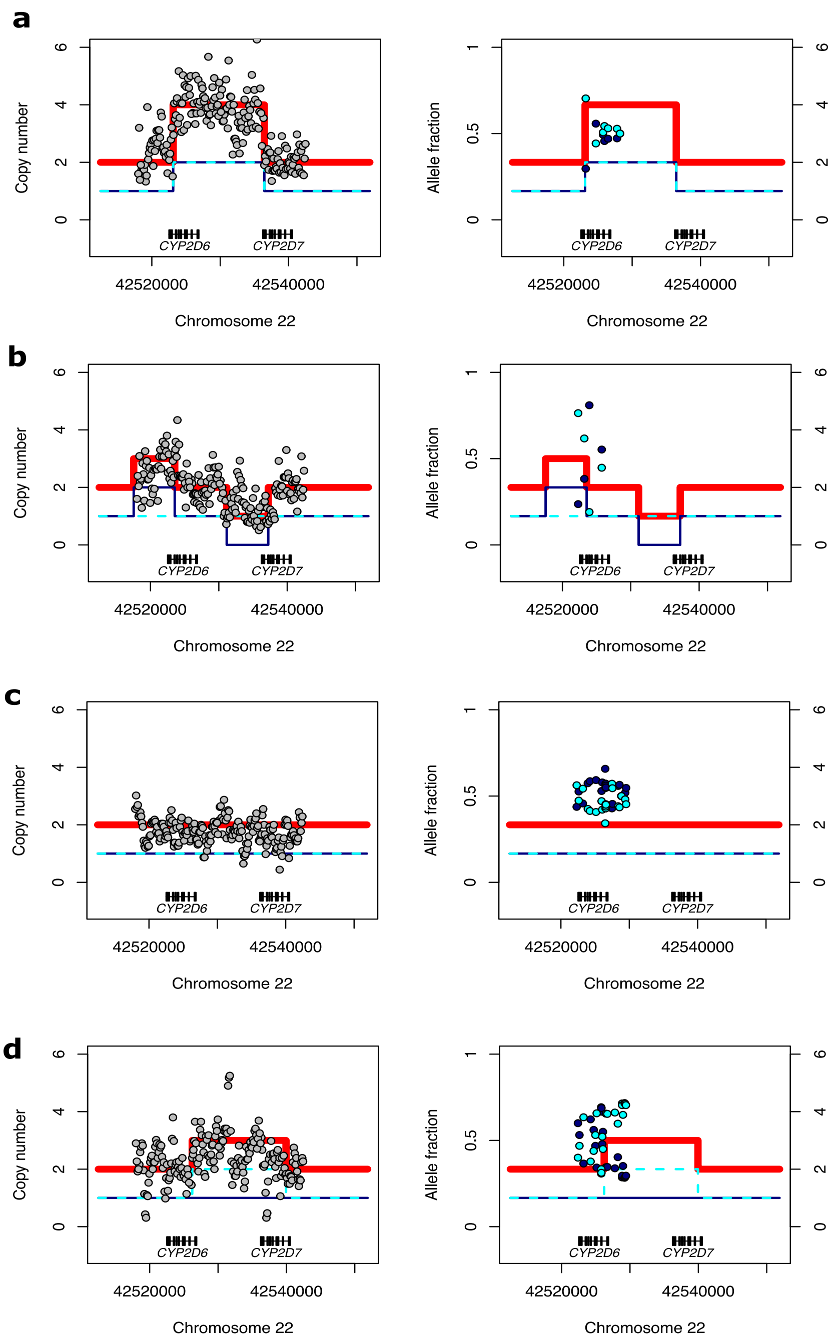

Examples of Stargazer plots for structural variants in Set 2. (a) Shows SIM119 with the *36+*10/*36+*10 diplotype. Notice the duplication and the exon 9 conversion deduced for both haplotypes (sky blue and navy blue dotted lines) (b) Shows SIM198 with a *CYP2D7/2D6* hybrid rearrangement (*13+*2). The other haplotype is *39. (c) Shows NA12892 with the **2/*3* diplotype (included for comparison). (d) Shows NA12878 with the **3/*68+*4* diplotype. Notice the *CYP2D6* to *CYP2D7* gene conversion from intron 1 onward shown by the dip in depth of coverage.

Supplementary Data Set 1

This table summarises the *CYP2D6* haplotypes used for creating diplotype simulations (Set 1 and Set 2). Detailed haplotype descriptions can be found at https://www.pharmvar.org/gene/CYP2D6.

Supplementary Data Set 2

Stargazer, Astrolabe, and Aldy results for simulated *CYP2D6* homozygous star alleles (N=154).

The improvement in allele calls by Aldy and Astrolabe after augmenting their databases with previously missing suballeles is reflected in columns labelled 'Run 1' and 'Run 2' respectively.

For Stargazer, we indicate cases where correct haplotype(s) are reported under hap1_cand or hap2_cand but not under hap1_main or hap2_main (hap1_main and hap2_main are used to calculate the activity score). Stargazer (v1.0.7) prefers to report background 'functionally relevant' alleles for samples with uncertain function alleles (e.g. reporting *17 instead of *58). This kind of reporting might have little effect on the phenotype prediction but it could lead to elevated frequencies of alleles such as *1, *2, *10 and *17 if Stargazer is used in population studies.

Supplementary Data Set 3

Main allele calls for simulated diplotype combinations (starting from BAM files) of haplotypes with definitive or moderate PharmVar level of evidence.

For Stargazer, the reported diplotypes are those that the tool considers as the main diplotypes (i.e. the ones it uses to calculate the activity score). The reference panel used for Stargazer's statistical phasing was the default 1000 genomes reference VCF.

For this part of the analysis, undefined suballeles were included without modifying current tool databases to simulate the scenario of a user using these tools on a novel WGS dataset. As such, some of the miscalls (especially for Astrolabe and Aldy) indicate the potential effect that novel suballeles in understudied populations could have on the accuracy of these algorithms. Stargazer (v1.0.7) does not call suballeles. Therefore it's difficult to ascertain the effect of novel suballeles on Stargazer's accuracy.

Some miscalls could also be due to sequencing noise in the simulated BAM files as this is also expected in real world NGS data especially for the *CYP2D* region.

Supplementary Data Set 4

Astrolabe, Aldy, and Stargazer calls for samples with simulated *CYP2D6* structural variants at 30x, 60x, and 100x.

Supplementary Data Set 5

Genotypes called by Astrolabe, Aldy, Stargazer and their ensemble for 75 real WGS samples in comparison to GeT-RM consensus calls.

Supplementary Data Set 6

A list of pharmacogenes that can be genotyped using Astrolabe (v0.8.7.0), Aldy (v2.2.3), and Stargazer (v1.0.7).
